# Supplementary material for: 2′-O-methylation-dependent installation of N2-methylguanosine in the U6 internal stem loop facilitates efficient spliceosome assembly
Source: Nat Commun. 2026 Apr 24;17:3793. doi: 10.1038/s41467-026-72355-2 (PMC13109388; doi:10.1038/s41467-026-72355-2)
Supplement: Supplementary file 1 — Supplementary Information [file 41467_2026_72355_MOESM1_ESM.pdf]

SUPPLEMENTARY INFORMATION

2′-*O*-methylation-dependent installation of *N*^{2}-methylguanosine in the U6 internal stem loop facilitates efficient spliceosome assembly

Nicole Kleiber, Jonny Petrosyan, Maria Greve, Chairini C. Thomé, Olexandr Dybkov, Laurianne L.E. Tay, Luisa M. Welp, Philipp Hackert, Holger Stark, Markus T. Bohnsack, Henning Urlaub, Lydia Herzel, Marc Graille, Claudia Höbartner, Katherine E. Bohnsack

SUPPLEMENTARY TEXT

Verification of synthesized RNAs

The purity of the synthesized RNA samples was analyzed by anion-exchange HPLC (Dionex DNAPac PA200, 2 × 250 mm, flow rate 0.5 mL/min, at 60 °C; Solvent A: 25 mM Tris-HCl (pH 8.0), 6 M Urea. Solvent B: 25 mM Tris-HCl (pH 8.0), 6 M Urea, 0.5 M $NaClO_4$. Gradient: 0‑48% solvent B in 12 CV). The resulting anion exchange HPLC-traces (260 nm) are shown below.

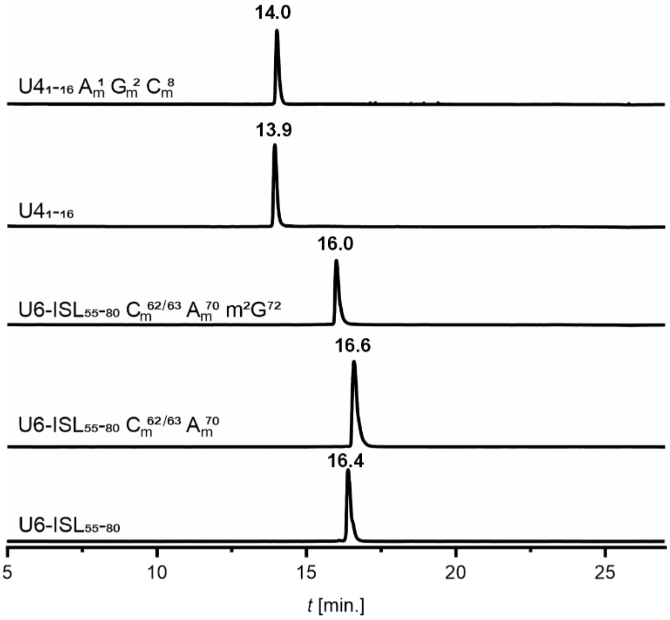

To confirm the identity of all synthesized RNA samples, HR-ESI-MS analysis on a Bruker micrOTOF-Q-III (negative ion mode, direct injection) was performed. The monoisotopic masses were obtained by charge deconvolution. The results are reported below.

Oligonucleotide	Sum Formula	Mass calculated [Da]	Mass found [Da]
U6-ISL$_{55-80}$	$C_{248}H_{310}N_{103}O_{177}P_{25}$	8336.18686	8336.21252
U6-ISL$_{55-80}$ Cm$_{62/63}$ Am$_{70}$	$C_{251}H_{316}N_{103}O_{177}P_{25}$	8378.23381	8378.25405
U6-ISL$_{55-80}$ Cm$_{62/63}$ Am$_{70}$ m^{2}G$_{72}$	$C_{252}H_{318}N_{103}O_{177}P_{25}$	8392.24946	8392.24627
U4$_{1-16}$	$C_{152}H_{190}N_{61}O_{111}P_{15}$	5109.76177	5109.73947
U4$_{1-16}$ Am^1 Gm^2 Cm^8	$C_{155}H_{196}N_{61}O_{111}P_{15}$	5151.76372	5151.79028

Establishment of methylation-sensitive DNAzymes

To label unmodified and modified U6-ISLs with fluorescein on the 3'-end, periodate oxidation was used. The reaction was performed in a total volume of 48 µL. RNA (5 nmol), sodium phosphate buffer (pH 7.5, 50 mM) and $NaIO_4$ (20 mM) were incubated for 10 min at 37 °C. After addition of Na_2SO_3 (83 mM), the mixture was incubated for another 5 min at 37 °C. Following addition of Flu-TSC (8 mM), the final mixture was incubated for 2 h at 37 °C. Stop solution was added and the labeled RNA was purified via a 15% PAA gel. After ethanol precipitation, the labeled RNA was used for DNAzyme cleavage kinetic assays.

For DNAzyme cleavage kinetic assays, 10 pmol of 3'-fluorescein labeled unmodified and modified U6-ISL was annealed to 100 pmol of DNAzyme (m^{2}G, Am$_{70}$ or Cm$_{62}$/Cm$_{63}$) in reaction buffer (m^{2}G and Am$_{70}$: 50 mM HEPES (pH 7.4), 400 mM KCl, 100 mM NaCl; Cm$_{62}$/Cm$_{63}$: 50 mM HEPES (pH 7.4), 150 mM NaCl). The reaction was initiated by addition of 5 mM $MgCl_2$ (m^{2}G), 10 mM $MgCl_2$ and 10 mM $MnCl_2$ (Am$_{70}$) or 1 mM $MgCl_2$ and 1 mM $MnCl_2$ (Cm$_{62}$/Cm$_{63}$). After incubation at 25 °C, 1 µL aliquots were quenched at the desired timepoints in 4 µL of stop solution. Subsequently, half of the reaction mixture was loaded on a 20% PAA gel. After running the gel for 1 h at 25 W, band intensities were visualized and analyzed *via* fluorescence imaging using a ChemiDOCMP with blue epi illumination and an 530/28 nm emission filter. All DNAzyme cleavage kinetic assays were performed in triplicates. To obtain k_{obs} the mean value of the cleaved fraction (%) for each time point were plotted against the respective incubation time and fitted according to the equation below where P_{max} = final yield using OriginPro2023b.

$$y = P_{max} * (1 - \exp(-k_{obs} * t))$$

The cleavage site for the Cm$_{62}$/Cm$_{63}$ DNAzyme was confirmed *via* HR-ESI-MS. The sum formula, calculated and found masses of the U6-ISL fragments after incubation with the Cm$_{62}$/Cm$_{63}$ DNAzyme for 4 h at 25 °C are shown below.

Oligonucleotide	Sum Formula	Mass calculated [Da]	Mass found [Da]
3'-fragment	$C_{164}H_{203}N_{71}O_{114}P_{16}$	5485.80774	5485.89581
5'-fragment	$C_{84}H_{107}N_{32}O_{63}P_{9}$	2850.37967	2850.43181

SUPPLEMENTARY FIGURES AND LEGENDS

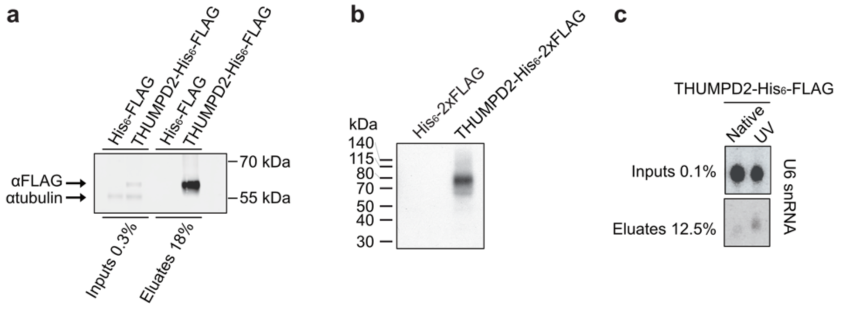

Supplementary Figure S1. Immunoprecipitation of THUMPD2-His$_6$-2xFLAG and protein–RNA crosslinking (a-b) HEK293 over-expressing His$_6$-2xFLAG or THUMPD2-His$_6$-2xFLAG were UV-crosslinked and used in n=2 anti-FLAG immunoprecipitation experiments. The FLAG-tagged versions of the proteins were detected by western blotting with an anti-FLAG antibody and tubulin was used as a loading control (a). After partial RNase digestion, the 5′ end of recovered RNA fragments were 5′-labelled with [^{32}P] and adaptors were ligated to the 5′ and 3′ ends. Protein–RNA complexes were separated by denaturing PAGE, transferred to a nylon membrane and detected by autoradiography (b). **(c)** Extracts from cells expressing THUMPD2-His$_6$-2xFLAG that had been crosslinked (UV) or not (Native) were used for anti-FLAG immunoprecipitation experiments (n=1). RNA was eluted from beads by Proteinase K treatment and RNAs in input and eluate samples were separated by denaturing PAGE and U6 snRNA was detected by northern blotting. Note the increased recovery of U6 after crosslinking and the upwards shift of the U6 signal, demonstrating successful protein–RNA crosslinking.

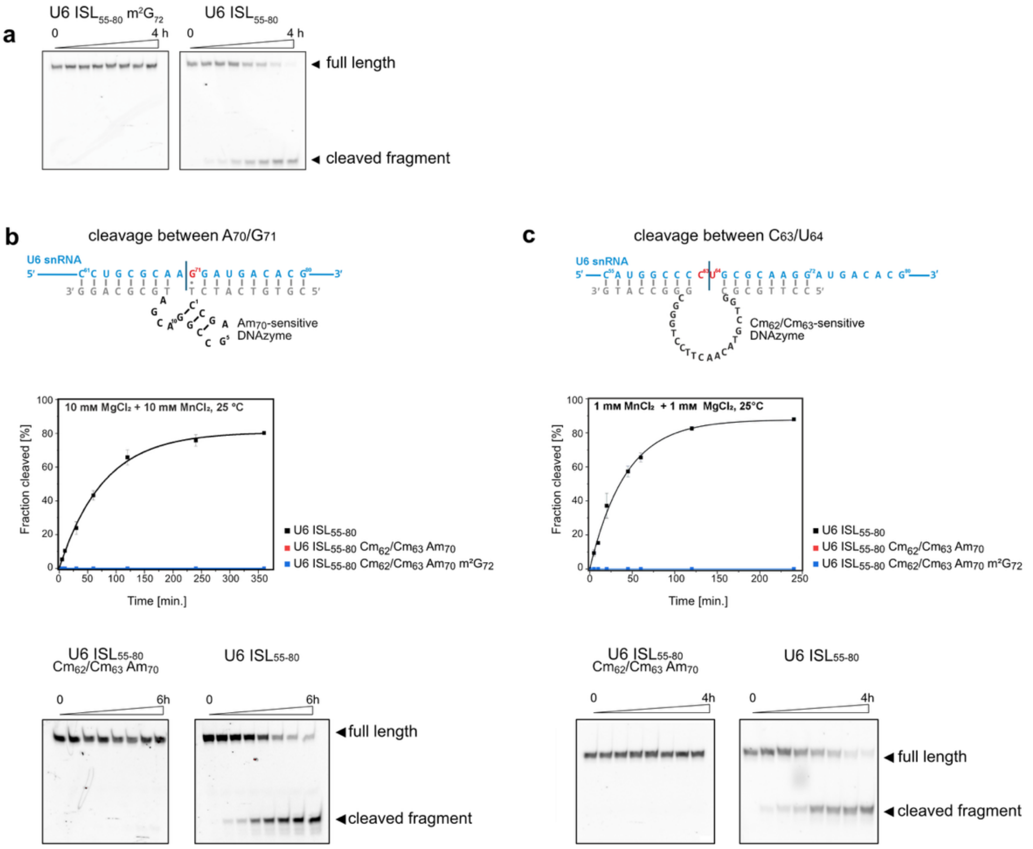

Supplementary Figure S2. Characterization of DNAzymes for the detection of U6 ISL modifications. (a) Synthetic U6 ISL_{55-80} m^2G-containing (left) or unmodified (right) was treated with an m^2G-sensitive DNAzyme that cleaves unmodified RNA. Formation of cleavage fragments was analyzed by denaturing PAGE for the timepoints indicated between 0 and 240 min. Arrows indicate full length RNA oligonucleotide or cleaved product. Representative gel images from n=3 independent experiments. **(b-c)** Schematic view of base-pairing of the Am_{70}- (b, top) or Cm_{62}/Cm_{63}-sensitive DNAzymes (c, top) with U6 snRNA (in blue) to detect the respective 2′-*O*-methylations. The cleavage site is indicated in the U6 sequence. As in (a), RNA oligonucleotides containing the sequence of the U6 ISL_{55-80} unmodified (black line) or containing the respective 2′-*O*-methylations (red and blue lines, left for Am_{70} and right for $Cm_{62}Cm_{63}$) were incubated with the respective DNAzymes and the fraction of cleaved RNA at the times indicated in the plots (middle panels) were determined after quantification of the bands shown in the gels (bottom panels). Arrows indicate full length RNA oligonucleotide or cleaved product. Data from n=3 independent experiments shown as mean ± standard deviation. Source data are provided as a Source Data file.

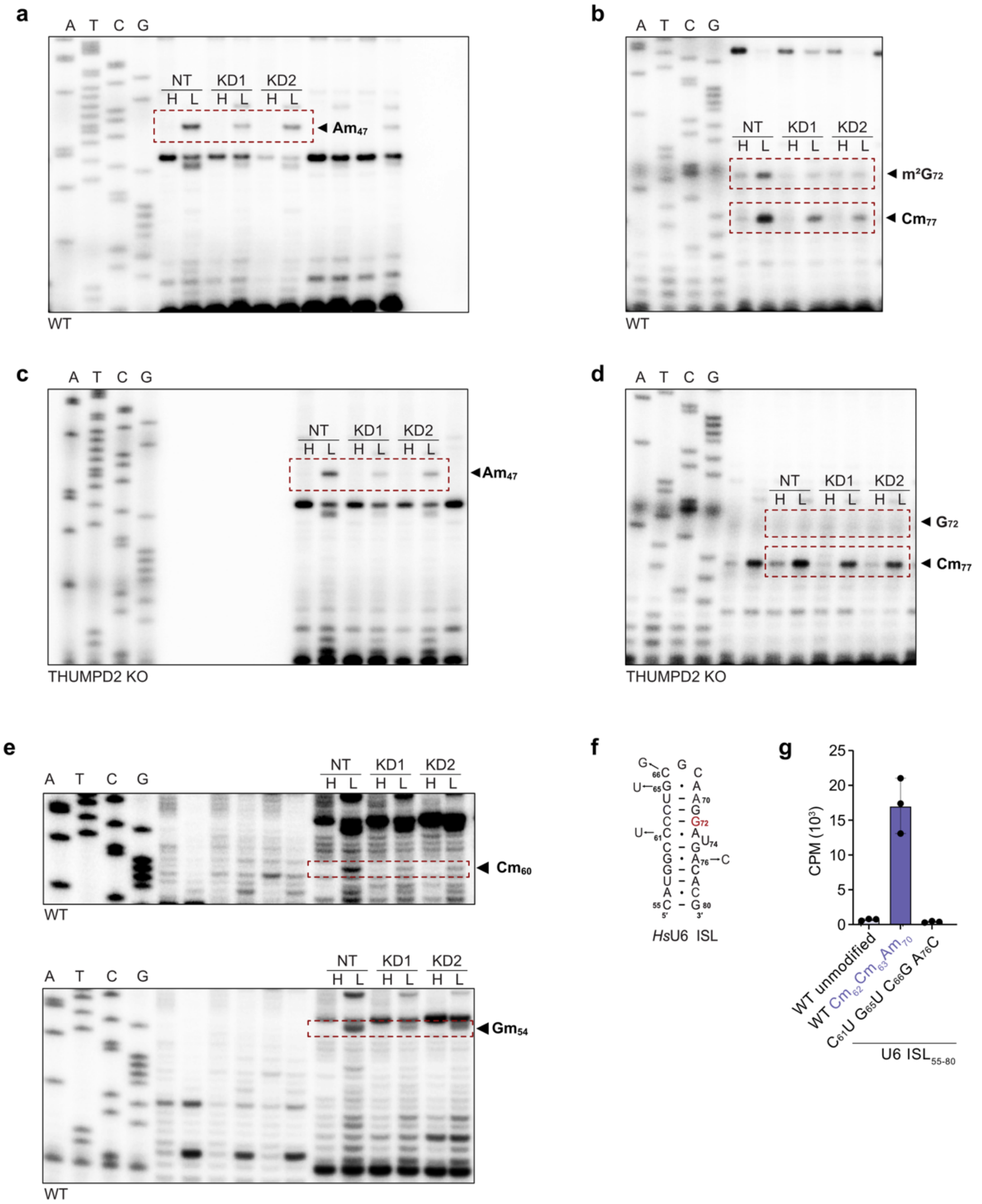

Supplementary Figure S3. LARP7-dependence of 2′-*O*-methylations in U6 determined by primer extensions and effect of U6 ISL stem stability on THUMPD2 methylation. (a-d) Uncropped primer extension gels represented in Fig 4h for RNA extracted from HCT116 WT (a, b) and THUMPD2 KO cells (c, d) after treatment with a non-target siRNA (NT) or siRNAs against LARP7 (KD1 and KD2). H = high dNTP concentration and L = low dNTP concentration. Red boxes highlight the cDNA fragments corresponding to the m^2G_{72} modification (b) and the fragments indicative of the presence of Cm_{77} (b,d) or Am_{47} (a,c). A sequencing ladder was run alongside each set of samples and A, T, C, G indicate ladders prepared using termination mixes containing ddATP, ddTTP, ddCTP and ddGTP, respectively.

Representative images for n=3 experiments. **(e)** RNA from siRNA-treated HCT116 WT cells was used for primer extension assays, as in (a-d) to detect Cm_{60} (top) and Gm_{54} (bottom). Representative image for n=2 experiments. **(f)** Schematic view of the human U6 ISL in which four sub-optimal base-pairs closest to G_{72} were strengthened by nucleotide substitutions ($C_{61}U$, $G_{65}U$, $C_{66}G$ and $A_{76}C$). **(g)** *In vitro* methylation assays were performed using purified His_6-ZZ-*Bt*THUMPD2-*Bt*TRMT112 protein complex, [^{3}H]-SAM as methyl group donor and the different RNA substrates, including WT unmodified and 2′-O-methylated U6 ISLs and the mutated ISL depicted in (f). Tritium incorporated in the substrate RNAs was quantified and bar plots show averaged counts per minute (CPM) of n=3 independent experiments with error bars representing mean ± standard deviation.

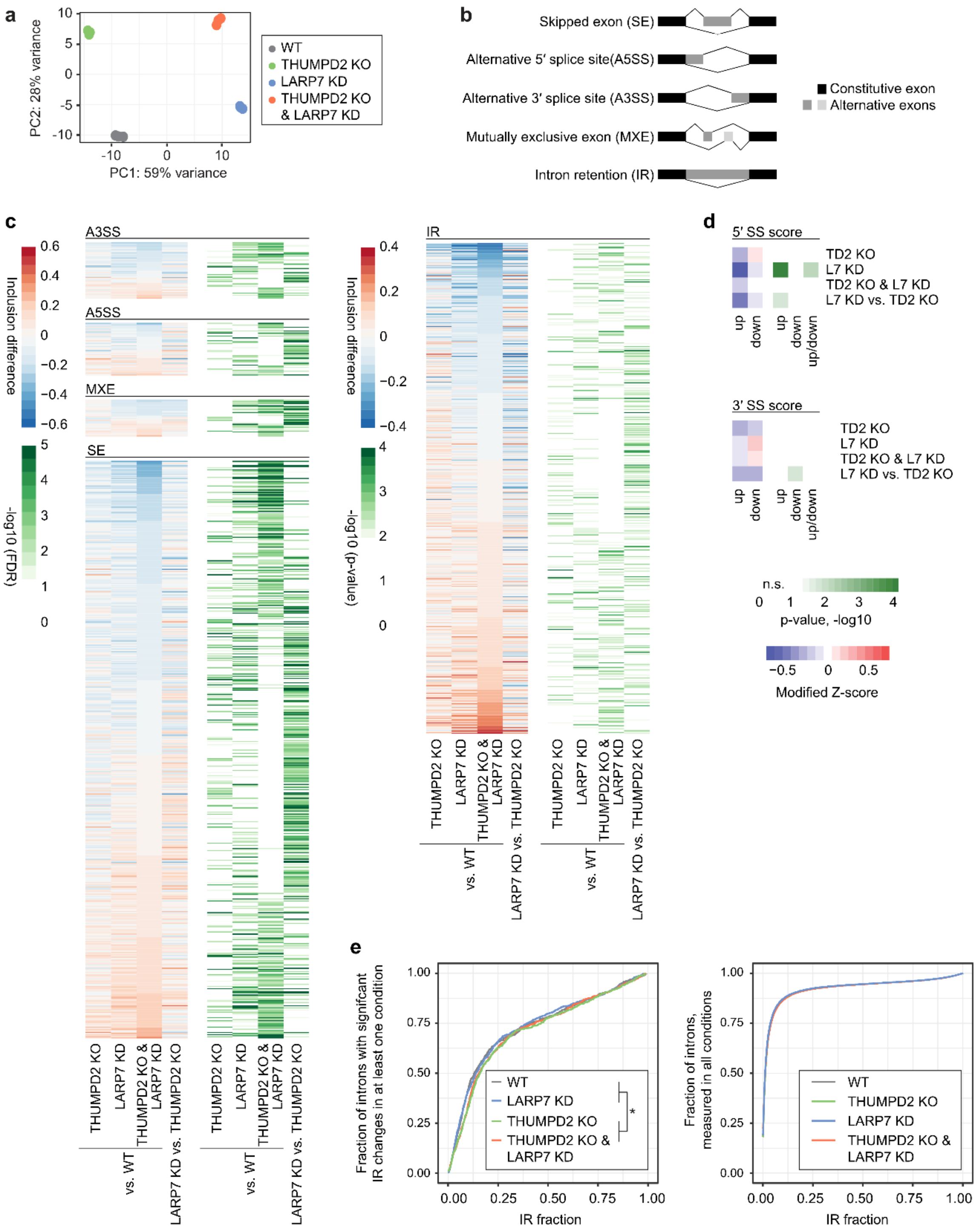

Supplementary Figure S4: **Characterization of RNA-seq datasets by principal component analysis and individual alternative splicing events. (a)** Principal component analysis based on gene-level expression quantification (top 5000 genes). The three biological replicates are shown as individual dots of the same color, and display good reproducibility. **(b)** Scheme of different types of alternative splicing. **(c)** Heatmap representation of alternative splicing events by AS type and sampled condition. Events are shown that change significantly in at least one of the 4 comparisons. Comparisons are made with respect to WT or between

LARP7 KD and THUMPD2 KO (right-most column). Significant AS events (rows) are sorted by increasing inclusion difference in the combined condition. Significant A3SS, A5SS, MXE and SE events are derived from rMATS (FDR < 0.01, absolute inclusion difference > 0.1, median read count in at least one condition > 50). Significant IR events are based on splicing efficiency calculation of annotated introns (p-value < 0.01, absolute inclusion difference > 0.05, median read count in at least one condition > 50). **(d)** Heatmaps showing the change of the median 5′/3′ splice site strength in more (up) or less (down) retained introns in the samples indicated. The modified Z-score relative to introns without significant changes and the significance of the changes plotted as -log10(p-value) from the Wilcoxon rank-sum test are plotted. LARP7 KD = L7 KD, THUMPD2 KO = TD2 KO. **(e)** Cumulative IR fraction distribution of introns that change pre-mRNA splicing significantly in at least one of the 4 comparisons (left) and of all introns passing the read count cutoff (right). p values of comparisons summarized with * were derived with the two-sided Kolmogorov-Smirnov with multiple testing correction using the Bonferroni method. WT against THUMPD2 KO 0.009792; WT against LARP7 KD & THUMPD2 KO 0.025404; LARP7 KD against THUMPD2 KO 0.021108; LARP7 KD against LARP7 KD & THUMPD2 KO 0.043554, and 1 for WT against LARP7 KD and THUMPD2 KO against LARP7 KD & THUMPD2 KO, respectively.

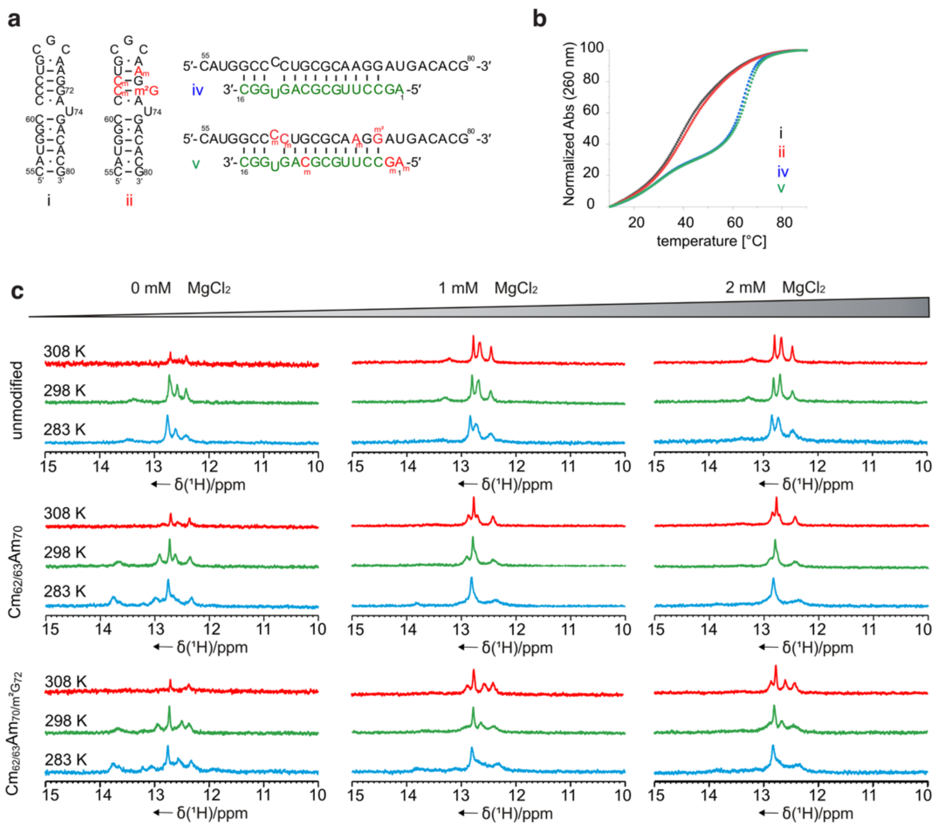

Supplementary Figure S5. Analyses of U4/U6 partial duplex and U6 ISL stability and structure *in vitro*, and snRNA levels in wild-type and THUMPD2 KO cells. (a) Schematic view of the synthetic RNA samples used for biophysical investigations. Modified nucleotides are shown in red. The U6 ISLs (i and ii) are as in Fig. 6a and within the modified and unmodified U4/U6 partial duplexes (iv and v), U6 is shown in black and U4 in green. **(b)** UV thermal melting curves for the samples shown in (a) plotting hyperchromicity at 260 nm. Conditions: 1 µM RNA, 100 mM NaCl, 10 mM potassium phosphate buffer pH 7.4, T = 10 °C – 90 °C. One of n=4 reversible heating and cooling curves is shown. **(c)** 1D ^{1}H-NMR spectra of imino region, 120 µM RNA in 10 mM phosphate buffer pH 7.4, 90% H_2O/ 10% D_2O at 283 K, 298 K or 308 K in the presence of 0, 1 or 2 mM $MgCl_2$.

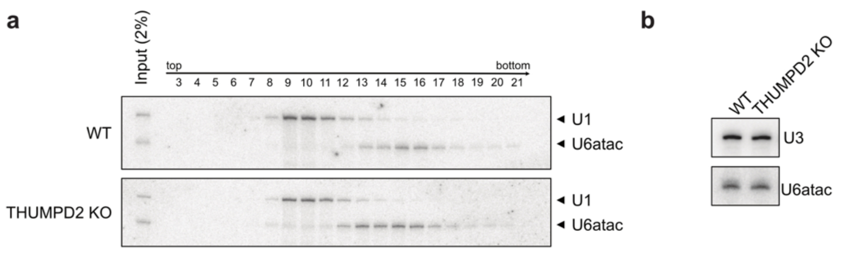

Supplementary Figure S6. Effect of THUMPD2 KO on U6atac levels and assembly into snRNPs. (a) Sedimentation of snRNP particles from HCT116 WT and THUMPD2 KO cells in a 10-30% glycerol gradient was analyzed by northern blotting using probes against U1 and U6atac. Representative data from n=3 experiments is shown. **(b)** Northern blot analysis of the levels of U6atac compared to the U3 snoRNA (loading control) in nuclear extracts from wild-type (WT) and THUMPD2 KO cells. Representative data from n=3 experiments is shown.

a

	Type of modification	Modification symbol and position	Enzyme or enzyme/guide RNA(s)	Source (PMID)
**Terminal modifications**	5′ Capping	5′ γ-monomethyl	MEPCE	8175647; 17643375
	3′ End extension and trimming	3′ terminal 2′,3′-cyclic phosphate	TUT1 and USB1	TUT1 (U6 TUTase): 9628908; 16790842. USB1 (Mpn1): 8995392; 22899009; 23022480; 23190533
**Internal modifications**	Pseudouridylations	Ψ31	Dyskerin / SNORA79 (ACA65)	SNORA79 (ACA65): 16373490
		Ψ40	Dyskerin / SCARNA23/3(ACA12)	SCARNA23 (ACA12): 15199136 and SCARNA3 (HBI-100): 11387227
		Ψ86	Dyskerin / SNORA79 (ACA65)	SNORA79 (ACA65): 16373490
	2′-*O*-methylations	Am47	Fibrillarin / SNORD7	SNORD7 (mgU6-47): 9844635
		Am53	Fibrillarin / SNORD8/SNORD9	SNORD8/9 (mgU6-53): 10490628
		Gm54	Fibrillarin / ?	-
		Cm60	Fibrillarin / SNORD67	SNORD67 (HBII-166): 11387227; 40316533
		Cm62	Fibrillarin / SNORD94	SNORD94 (U94-U106): 14602913
		Cm63	Fibrillarin / ? (possibly SNORD88A/B)	SNORD88A/B: 37792516
		Am70	Fibrillarin / ? (possibly SNORD23)	SNORD23: 40001124
		Cm77	Fibrillarin / SNORD10	SNORD10 (mgU6-77): 9844635
	Base methylations	m^6A_{43}	METTL16	28525753; 29051200
		m^2G_{72}	THUMPD2	37283053; 38165050; this study

b

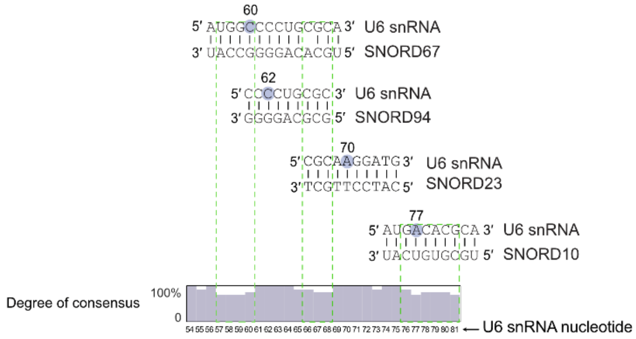

Supplementary Figure S7. U6 snRNA modifications and snoRNA base-pairings within the U6 ISL. (a) Summary of the mapped U6 snRNA terminal (5′ and 3′) and internal modifications (pseudouridylations, 2′-*O*-methylations and base methylations). Enzymes catalyzing the indicated modifications are listed on the right and where applicable guide RNAs are indicated. Source publications (PMID) are indicated on the right. **(b)** Base-pairings between the U6 ISL and box C/D snoRNAs guiding the 2′-*O*-methylation of C_{60}, C_{62}, A_{70} and A_{77} are shown. 2′-*O*-methylated nucleotides are highlighted and the position in the U6 sequence is indicated. Regions boxed in a dashed green line correspond to those with lower conservation scores as indicated in the bar plot representing the degree of consensus (0 = no conservation; 100% = maximum percentage identity) from Fig. 4k.
