# Supplementary material for: 2′-O-methylation-dependent installation of N2-methylguanosine in the U6 internal stem loop facilitates efficient spliceosome assembly
Source: Nat Commun. 2026 Apr 24;17:3793. doi: 10.1038/s41467-026-72355-2 (PMC13109388; doi:10.1038/s41467-026-72355-2)
Supplement: Supplementary file 2 — Description of Additional Supplementary Files [file 41467_2026_72355_MOESM2_ESM.pdf]

Description of Additional supplementary files

Supplementary Data 1: Output parameters of CRAC data mapping and overview of the number of sequencing reads mapping to different classes of RNA in the His6- 2xFLAG and THUMPD2- His6-2xFLAG CRAC datasets.

Supplementary Data 2: Raw data of U6 snRNA nucleoside quantification in U6 snRNA from WT and THUMPD2 KO cells.

Supplementary Data 3: Overview of the number of normalized sequencing reads mapping to the U6 snRNA precursor sequence in the His6-2xFLAG and THUMPD2-His6-2xFLAG 3' RACE datasets.

Supplementary Data 4: Nuclear proteins identified by DIA-MS in the THUMPD2- His6-2xFLAG IP with UVcrosslinking.

Supplementary Data 5: RNA contact sites mapped in THUMPD2 after RNA-protein crosslinking and mass spectrometry.

Supplementary Data 6: Differential gene expression quantified by DEseq2 of WT, THUMPD2 KO, LARP7 KD and the combined THUMPD2 KO & LARP7 KD.

Supplementary Data 7: Significant AS events derived from rMATS analysis.

Supplementary Data 8: Significant AS events derived from IR analysis and IR fractions.

Supplementary Data 9: DNA oligonucleotides used in this study.

Supplementary Data 10: Plasmids used in this study.

Supplementary Data 11: Antibodies used in this study.

Supplementary Data 12: RNA oligonucleotides used in this study.

Supplementary Data 13: siRNAs used in this study.
