# Supplementary material for: 2′-O-methylation-dependent installation of N2-methylguanosine in the U6 internal stem loop facilitates efficient spliceosome assembly
Source: Nat Commun. 2026 Apr 24;17:3793. doi: 10.1038/s41467-026-72355-2 (PMC13109388; doi:10.1038/s41467-026-72355-2)
Supplement: Supplementary file 16 — Reporting Summary [file 41467_2026_72355_MOESM16_ESM.pdf]

nature portfolio

Corresponding author(s): Katherine E. Bohnsack

Last updated by author(s): 2026/03/28

Reporting Summary

Nature Portfolio wishes to improve the reproducibility of the work that we publish. This form provides structure for consistency and transparency in reporting. For further information on Nature Portfolio policies, see our Editorial Policies and the Editorial Policy Checklist.

Statistics

For all statistical analyses, confirm that the following items are present in the figure legend, table legend, main text, or Methods section.

n/a	Confirmed	
☐	☒	The exact sample size (*n*) for each experimental group/condition, given as a discrete number and unit of measurement
☐	☒	A statement on whether measurements were taken from distinct samples or whether the same sample was measured repeatedly
☐	☒	The statistical test(s) used AND whether they are one- or two-sided<br>*Only common tests should be described solely by name; describe more complex techniques in the Methods section.*
☒	☐	A description of all covariates tested
☐	☒	A description of any assumptions or corrections, such as tests of normality and adjustment for multiple comparisons
☐	☒	A full description of the statistical parameters including central tendency (e.g. means) or other basic estimates (e.g. regression coefficient) AND variation (e.g. standard deviation) or associated estimates of uncertainty (e.g. confidence intervals)
☐	☒	For null hypothesis testing, the test statistic (e.g. *F*, *t*, *r*) with confidence intervals, effect sizes, degrees of freedom and *P* value noted<br>*Give P values as exact values whenever suitable.*
☒	☐	For Bayesian analysis, information on the choice of priors and Markov chain Monte Carlo settings
☒	☐	For hierarchical and complex designs, identification of the appropriate level for tests and full reporting of outcomes
☒	☐	Estimates of effect sizes (e.g. Cohen's *d*, Pearson's *r*), indicating how they were calculated

Our web collection on statistics for biologists contains articles on many of the points above.

Software and code

Policy information about availability of computer code

Data collection	ImageStudio Lite version 5.2.5
Data analysis	Softwares used<br>R (v4.3.2 and v4.4.0)<br>GraphPad Prism (v9)<br>Image Studio (v5.2.5, LI-COR)<br>PEAR (v0.9.6)<br>Fiji (v2.14.0)<br>ImageJ (v2.14.0)<br>pyCRAC (v1.4.5 and v1.5.2)<br>Flexbar (v3.5.0)<br>STAR (v2.7.10a and v2.7.11b)<br>igvtools (v2.12.3)<br>PEAKachu (v 0.2.0)<br>DESeq2 package (v1.42.1 and v1.48.2)<br>featureCounts (v2.0.0)<br>Bowtie2 (v2.3.5.1 and v2.5.3)<br>FastQC (v0.11.4)<br>Perl (v5.32.1)<br>SAMtools (v1.19 and v1.9)<br>BAMtools (v2.5.2)

Spectronaut (v18.3, Biognosys)
OpenMS pipeline NuXL (v1)
OpenMS TOPPViewer (v2.6.0)
AlphaFold (v3)
bedSort (v469)
bedGraphToBigWig (v469)
BEDTools (v2.27.1)
rMATS-turbo (v4.3.0)
maser (v1.26.0)
pheatmap (v1.0.13)
ggplot2 (v4.0.1 and v3.5.2)
UpSetR (v1.4.0)
ComplexUpset (v1.3.6)
ggsashimi (v1.1.5)
TopSpin (v3.5 pl7)
TopSpin (v4.3.0)
biomaRt (v2.64.0)
GenomicFeatures and GenomicRanges (v1.60.0)
AnnotationDbi (v1.70.0)
Biobase (v2.68.0)
GenomeInfoDb (v1.44.3)
IRanges (v2.42.0)
tibble (v3.3.0)
gridExtra (v2.3)
ggpubr (v0.6.2)
RColorBrewer (v1.1-3)
dplyr (v1.1.4)
tidyr (v1.3.1)
MikroWin 300 SL (v5.63)
ImageQuant (v8.1)
Typhoon FLA9500 Control Software (v1.0)
FluorEssence Software (v3.9)
Leica Application Suite X (v4.6.1.27508)
MAFFT (v7)

Code availability
The code developed for optimized intron retention analysis is available via GitHub [https://doi.org/10.5281/zenodo.18197506].

For manuscripts utilizing custom algorithms or software that are central to the research but not yet described in published literature, software must be made available to editors and reviewers. We strongly encourage code deposition in a community repository (e.g. GitHub). See the Nature Portfolio guidelines for submitting code & software for further information.

Data

Policy information about availability of data

All manuscripts must include a data availability statement. This statement should provide the following information, where applicable:

- Accession codes, unique identifiers, or web links for publicly available datasets
- A description of any restrictions on data availability
- For clinical datasets or third party data, please ensure that the statement adheres to our policy

The deep sequencing data generated during this study have been deposited in Gene Expression Omnibus (GEO) database [http://www.ncbi.nlm.nih.gov/geo/] under the following accession codes:
GSE270752 [https://www.ncbi.nlm.nih.gov/geo/query/acc.cgi?acc=GSE270752]: CRAC datasets for THUMPD2-His6-2xFLAG and the His6-2xFLAG tag.
GSE270753 [https://www.ncbi.nlm.nih.gov/geo/query/acc.cgi?acc=GSE270753]: 3′ RACE datasets for THUMPD2-His6-2xFLAG and the His6-2xFLAG tag.
GSE303357 [https://www.ncbi.nlm.nih.gov/geo/query/acc.cgi?acc=GSE303357]: RNA-seq datasets for WT and THUMPD2 KO treated with siNT or siLARP7_1.

The proteomics data generated during this study have been deposited at the ProteomeXchange Consortium via the PRIDE partner repository [https://www.ebi.ac.uk/pride/] under the following accession code and submission reference:
PXD053510: IP-MS datasets for THUMPD2-His6-2xFLAG and the His6-2xFLAG tag.
PXD053619: Protein-RNA cross-linking MS dataset for THUMPD2-His6-2xFLAG.

Source data are provided with this paper.

Research involving human participants, their data, or biological material

Policy information about studies with human participants or human data. See also policy information about sex, gender (identity/presentation), and sexual orientation and race, ethnicity and racism.

Reporting on sex and gender	Not applicable.
Reporting on race, ethnicity, or	Not applicable.

other socially relevant groupings	
Population characteristics	Not applicable.
Recruitment	Not applicable.
Ethics oversight	Not applicable.

Note that full information on the approval of the study protocol must also be provided in the manuscript.

Field-specific reporting

Please select the one below that is the best fit for your research. If you are not sure, read the appropriate sections before making your selection.

☒ Life sciences ☐ Behavioural & social sciences ☐ Ecological, evolutionary & environmental sciences

For a reference copy of the document with all sections, see nature.com/documents/nr-reporting-summary-flat.pdf

Life sciences study design

All studies must disclose on these points even when the disclosure is negative.

Sample size	Statistical determination of sample size was not performed. In the case of non-quantitative experiments, sample size was determined to demonstrate clear reproducibility of the results (up to three independent experiments). Where the outcomes of experiments were quantified, at least three independent replicates were performed and statistical analyses of the data were done using appropriate statistical tests (detailed in the Methods section and Figure Legends).
Data exclusions	Data derived from experiments in which technical or human errors arose were excluded. Exclusion criteria were not pre-established. Exclusions were based on observations during the experimental procedures and the quality of the generated data.
Replication	Reproducibility was determined by replications and statistical analyses where appropriate. The number of replicates of each experiment presented in each figure are given in the figure legend. Once robust protocols were established, data generated in experiments without technical errors were highly reproducible.
Randomization	Randomization was not performed during biochemical and cell biological experiments. Covariates were controlled by growing cells under standardized conditions and utilizing standardized extraction techniques for purifying components e.g. proteins, RNAs, etc.
Blinding	Blinded experiments were not performed in this study. Experiments mostly compared specific treatments of otherwise comparable samples. To conduct the experiments, it was necessary for the investigators to be aware of the treatment/condition applied. Appropriate cellular and biochemical controls were included in each experiment/replicate.

Reporting for specific materials, systems and methods

We require information from authors about some types of materials, experimental systems and methods used in many studies. Here, indicate whether each material, system or method listed is relevant to your study. If you are not sure if a list item applies to your research, read the appropriate section before selecting a response.

Materials & experimental systems

n/a	Involved in the study	
☐	☒	Antibodies
☐	☒	Eukaryotic cell lines
☒	☐	Palaeontology and archaeology
☒	☐	Animals and other organisms
☒	☐	Clinical data
☒	☐	Dual use research of concern
☒	☐	Plants

Methods

n/a	Involved in the study	
☒	☐	ChIP-seq
☒	☐	Flow cytometry
☒	☐	MRI-based neuroimaging

Antibodies

Antibodies used	See Supplementary Data 11.
Validation	Antibodies were validated by western blotting. Signals corresponding to the appropriate sizes of the target proteins were detected (see Figs. 1f, 3i, l, 4g, 6i-l, S1a).<br>The antibodies against THUMPD2 and LARP7 were validated in this study by comparing extracts from RNAi-treated cells or cells genomically deleted of target genes (see Figs. 3l and 4g). The THUMPD2 antibody has previously been used in PMID: 37283053. The LARP7 antibody was KO/KD validated by the manufacturer and has been used in 13 peer-reviewed publications listed on the

manufacturer’s website (https://www.ptglab.com/products/LARP7-Antibody-17067-1-AP.htm?srsltid=AfmBOoq0O6pWdFRuy9F1MDxThGA4aA6fnuvv2PTokbTf1atk0p-LdCvS#publications).
To validate the anti-FLAG antibody, extracts from HEK293 cells expressing or not expressing tagged versions of specific proteins were analyzed (see Figs1f, 3i, l). This antibody has been used in 8220 peer-reviewed manuscripts cited on the manufacturer's website (https://www.sigmaaldrich.com/DE/en/search/f3165?focus=papers&page=1&perpage=30&sort=relevance&term=F3165&type=citation_search).

All other antibodies used for western blotting in this study have previously been used in peer-reviewed publications listed on the manufacturer’s websites:
Anti-Tubulin (Rabbit): (https://www.ptglab.com/products/TUBA1B-Antibody-11224-1-AP.htm?srsltid=AfmBOorEpYG1OLgagVIjOpjKjK2MUPQDInjrGL3TU5fse_-WBGiuokVZ#publications)
Anti-Fibrillarin: (https://www.scbt.com/p/fibrillarin-antibody-g-8?srsltid=AfmBOoqj7xkN1UKN1q0u4mfLl-5gQ9O-MvEojelJVPtmLaM8TaNK5roi)
Anti-TRMT112: https://www.scbt.com/p/trmt112-antibody-f-7?srsltid=AfmBOopzHUlwliOxWOPTKDIwSpK7-LTYNtV50UBRUstmtJLe51w4hzWR)
Anti-EFTUD2: (https://www.fortislife.com/products/primary-antibodies/rabbit-anti-eftud2-snrp116-antibody/BETHYL-A300-957)
Anti-MEPCE: (https://www.ptglab.com/products/MEPCE-Antibody-14917-1-AP.htm?srsltid=AfmBOopgzsicLAjEdzJdWPadw4JRKFd53RUV_fd2Srmcd6d8WjplEMr8)
Anti-PRPF4: (https://www.ptglab.com/products/PRPF4-Antibody-10728-1-AP.htm?srsltid=AfmBOooMkQLZxoZHDIpHU_hZXko8c6SBRpAowmbCcwh6M1l5sbyy4BpB)
Anti-PRPF3: (https://www.ptglab.com/products/PRPF3-Antibody-10106-1-AP.htm?srsltid=AfmBOoqF-gEJCeFJngTnrMPaauNNMl1kzc-EBZlG_3But-xk2itfkptz)
Anti-Tubulin (Mouse): (https://www.sigmaaldrich.com/DE/en/product/sigma/t6199?srsltid=AfmBOoqkL4XQ_StCSTXBafQE3IRGOJinvJyyJI3WdJDXABoeZkEIJb_6)

Antibodies used for immunfluorescence have been previously verified:
Anti-UTP14A: PMID: 38065954
Anti-SART3: PMID: 38985674

Eukaryotic cell lines

Policy information about cell lines and Sex and Gender in Research

Cell line source(s)	Cell lines used in this study are based on the HEK293 Flp-In T-Rex cell line (ThermoFisher Scientific; cat. no R78007) or HCT116 (ECACC; cat no 91091005).
Authentication	CRISPR-Cas-mediated genome disruption of THUMPD2 was confirmed by sequencing of genomic DNA, RNA-seq and western blotting (PMID: 37283053). Appropriate integration of cassettes for inducible expression of GFP-THUMPD2, THUMPD2-His6-2xFLAG or their derivatives were verified by western blotting (see Figs. 1f, 2a,b,e,f, 3g,h,i,l and S1a), and cells were maintained under appropriate antibiotic-mediated selection.
Mycoplasma contamination	Cell lines were routinely checked for mycoplasma contamination and confirmed to be negative.
Commonly misidentified lines (See ICLAC register)	No commonly misidentified cell lines were used in this study.

Plants

Seed stocks	Not applicable.
Novel plant genotypes	Not applicable.
Authentication	Not applicable.
